# Migration of foreign body from postcricoid region to the subcutaneous tissue of the neck

**DOI:** 10.4103/0256-4947.70565

**Published:** 2010

**Authors:** Mohd Mokhtar Shaariyah, Husain Salina, Banarsidas Dipak, Md Nasir Abd Majid

**Affiliations:** a From the Department of Otorhinolaryngology, Faculty of Medicine, Universiti Kebangsaan Malaysia Medical Centre, Kuala Lumpur, Malaysia; b From the General Hospital, Kuala Lumpur, Malaysia

## Abstract

Migration of a foreign body from the hypopharynx to the subcutaneous tissue of the neck is a rare event. We report a case of a 48-year-old male who accidentally swallowed a fish bone which was not identified intraoperatively. The patient then presented with migration of the bone to the soft tissue of the neck. We conclude that careful assessment of the patient with a foreign body in the throat is crucial to avoid fatal complications.

Fish bones are the most commonly ingested foreign body. They have a tendency to stick in the pharynx and mucosal layer of the gastrointestinal tract due to their linear shape and sharp edges, and can then cause serious complications if left untreated. In most cases, fish bones can be removed safely by endoscopy, but they may migrate extraluminally to the skin in rare cases.^1^ In our case the fish bone was not identified intraoperatively and it migrated from the postcricoid region to the subcutaneous tissue of the neck.

## CASE

**Figure 1:**
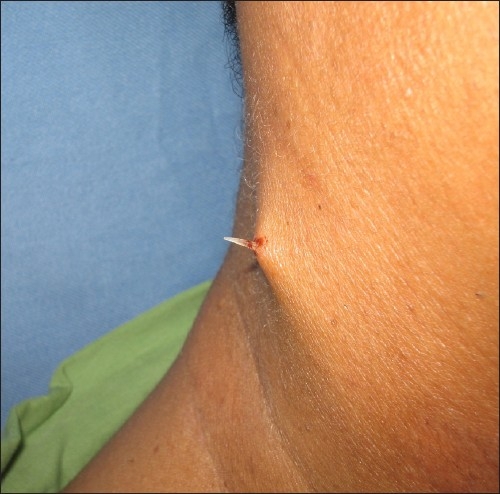
The fish bone protruding out of the right side of the neck.

**Figure 2:**
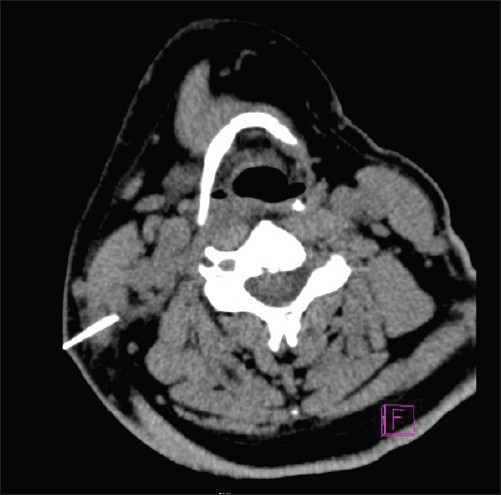
CT revealing the fish bone transversely located at the right sternocleidomastoid muscle.

**Figure 3:**
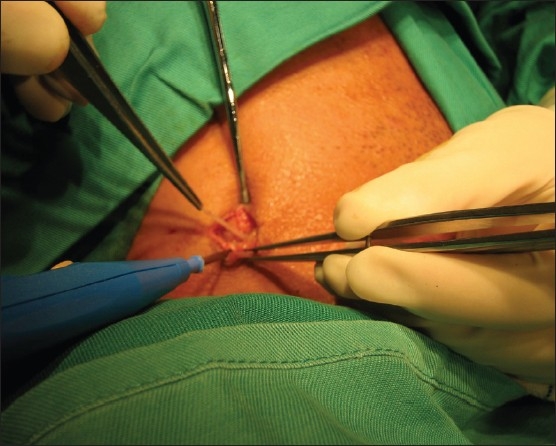
Intraoperative finding revealed the fish bone embedded in the subcutaneous tissue of the neck.

**Figure 4:**
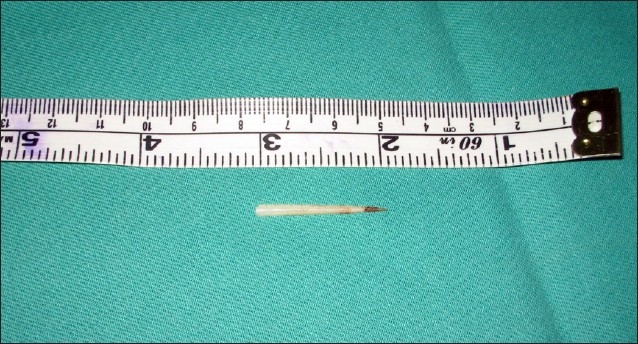
The fish bone after removal, showing linear shape with sharp edge.

A 46-year-old male presented to us with odynophagia following ingestion of a fish bone on the same day. Examination of the oral cavity revealed a fish bone stuck in the postcricoid region. An attempt to remove the fish bone under local anesthesia was unsuccessful because the patient had excessive gagging. The patient was then scheduled for direct laryngoscopy and esophagoscopy and removal of the foreign body under general anesthesia. The case was operated on 24 hours later due to heavy demand for emergency operation theater rooms. An ulcerated area was noted intraoperatively in the postcricoid region with no evidence of the embedded fish bone. The patient was discharged on the next day, but two days after the discharge, he developed a swelling in the right side of the neck accompanied by a little pain. He did not seek treatment until eight days later when he noticed the fish bone protruding out of his neck. Examination revealed the fish bone at the lateral border of the neck with no signs of any abscess (**Figure 1**). A CT scan showed the fish bone to be transversely located at the right sternocleidomastoid muscle (**Figure 2**). An emergency exploration of the neck was performed and the fish bone was gently pulled out of the right subcutaneous tissue of the neck (**Figure 3**); it was approximately 1 centimeter in length (**Figure 4**). The patient was discharged on the next day with a seven-day course of antibiotics. On subsequent clinical follow-up, the patient was asymptomatic.

## DISCUSSION

Ingested foreign bodies are one of the most common problems encountered by medical officers in the casualty ward or the otorhinolaryngology clinic. Most of the time, the foreign body is impacted at the tonsil or the base of tongue.^2^ An unidentified foreign body will usually spontaneously pass out through the alimentary tract without any complication. Spontaneous passage is usually related to the size and type of the foreign body.^3^ Fish bones are the most commonly ingested foreign body in Asian countries.^1^ They have a tendency to stick in the pharynx and mucosal layer of the gastrointestinal tract due to their linear shape and sharp edges, and can then cause serious complications if left untreated.

Migration of ingested foreign bodies is very rare.^1^,^5^ Twenty-four patients were reported with migration of foreign bodies within six years in Singapore.^1^ The largest reported series was of 1088 cases of foreign body ingestion in China.^3^ Most of the foreign bodies were reported to be lodged in the pharynx, esophagus, stomach, and duodenum. A fish bone has the ability to migrate to the thyroid gland, carotid artery, mediastinum, or subcutaneous tissue due to the constant contraction and relaxation of the pharyngeal musculature, esophageal peristalsis, and surrounding tissue reactions.^6^–^9^

Once a fish bone is stuck in the throat, it must be removed as soon as possible because of the higher likelihood of its migration due to its linear and sharply pointed contours. In this case, the removal of the foreign body was delayed and the patient was operated on almost 24 hours after the foreign body ingestion. No foreign body could be seen intraoperatively even after putting gentle pressure on the surrounding mucosal layer. We initially thought that the fish bone was dislodged into the gastrointestinal tract, but the patient returned with the fish bone protruding out through the subcutaneous tissue of the neck nine days after the first surgery. Studies have shown that it can take up to 41 days for a fish bone to be excreted naturally from the larynx or the gastrointestinal tract through the skin.^6^

Although plain radiography is a simple and valuable investigation to demonstrate the presence of an opaque foreign body, CT scanning helps to detect the exact site of the impacted foreign body and thus allows for predicting and avoiding any possible complications, especially in the case of migration. In our case, it was necessary to do the CT scan prior to the second surgery to assess the location of the impacted foreign body and its relation to the vital structures of the neck and avoid other possible fatal complications. An extraluminal foreign body has the potential to cause serious complications such as esophageal perforation, periesophageal abscess, retropharyngeal abscess, mediastinitis, arterioesophageal fistula, tracheoesophageal fistula, and carotid rupture. We managed to retrieve the fish bone from the subcutaneous tissue by making an adequate incision in the skin after confirmation of the location of the fish bone.

In conclusion, foreign body ingestion should be managed as soon as possible to avoid fatal and serious complications. It is important to carefully reassess the patient with a foreign body in the throat, especially a fish bone, which has not been found or has been missed by oral or endoscopic examination under general anesthesia.
